# An Incident of Practice

**Published:** 1884-03

**Authors:** C. E. Francis

**Affiliations:** New York


### AN INCIDENT OF PRACTICE.

BY C. E. FRANCIS, D. D. S., NEW YORK.

One afternoon, some five years ago, a lady residing in the neighborhood came rushing into my office, bringing with her a child—a little girl scarcely four years of age, who, but a few minutes before, was precipitated from a chair on which she had been standing, headlong to the floor. In her descent her left superior central incisor collided with the base of a walnut dressing case, and in an instant the tooth disappeared. The child's lip was cut and swollen, and the blood was flowing copiously. Wet napkins were applied, and after the hemorrhage was sufficiently checked for examination, it was found that the tooth still remained, but had been driven so far upwards that the coronal cutting edge extended beyond the line of the alveolar margin.

The following morning nitrous oxide was administered and the tooth carefully brought down to its original position. Topical treatment soon allayed the inflammation, and in time the tooth became firm.

Some degree of anxiety was felt concerning the germ of the permanent incisor, fearing that it might have suffered injury from the sudden and severe shock. In proper time, however, it made its appearance, bearing no evidence of having ever been disturbed.

